# Prognostic Value of the C-Reactive Protein–Albumin–Lymphocyte (CALLY) Index for 1-Year Mortality After Transcatheter Aortic Valve Implantation

**DOI:** 10.3390/jcdd13020083

**Published:** 2026-02-09

**Authors:** Hakan Süygün, Zeynep Seyma Turinay Ertop, Melike Polat, Murat Can Güney, Hüseyin Ayhan, Telat Keleş, Engin Bozkurt

**Affiliations:** 1 Department of Cardiology, Faculty of Medicine, Karamanoğlu Mehmetbey University, Karaman Training and Research Hospital, 70200 Karaman, Turkey; 2 Department of Cardiology, Medicana International Ankara Hospital, 06520 Ankara, Turkey; 3 Department of Cardiology, Faculty of Medicine, Atılım University, Medicana International Ankara Hospital, 06836 Ankara, Turkey; 4 Department of Cardiology, Faculty of Medicine, University of Health Sciences, Sincan Education and Research Hospital, 06018 Ankara, Turkey; 5 Department of Cardiology, Faculty of Medicine, Ankara Yıldırım Beyazıt University, Ankara City Hospital, 06800 Ankara, Turkey

**Keywords:** transcatheter aortic valve implantation, CALLY index, immunonutrition, inflammation, 1-year mortality

## Abstract

Objectives: Systemic inflammation, malnutrition, and immune dysregulation have emerged as important determinants of long-term outcomes after transcatheter aortic valve implantation (TAVI). The C-reactive protein–albumin–lymphocyte (CALLY) index is a novel immunonutritional biomarker that integrates these pathophysiological domains; however, its prognostic value in TAVI patients has not yet been investigated. This study aimed to evaluate the association between the CALLY index and 1-year mortality after TAVI. Methods: This retrospective observational study included 532 consecutive patients who underwent TAVI at a tertiary-care center between 2014 and 2023. Baseline laboratory parameters were obtained before the procedure, and the CALLY index was calculated as (albumin × lymphocyte count)/(C-reactive protein × 10). The primary endpoint was 1-year mortality. Receiver operating characteristic (ROC) curve analysis was performed to assess the discriminative ability of the CALLY index and conventional surgical risk scores. Multivariable regression analyses were used to identify independent predictors of mortality. Results: During the 1-year follow-up period, 85 patients (15.9%) died. Patients who died had significantly lower baseline CALLY index values compared to survivors (*p* < 0.001). The CALLY index demonstrated good discriminative performance for 1-year mortality (AUC: 0.797), outperforming EuroSCORE II (AUC: 0.705) and the Society of Thoracic Surgeons (STS) score (AUC: 0.619). A CALLY cut-off value of 0.45, derived using Youden’s index, was associated with a more than threefold increased risk of mortality. In multivariable analysis, the CALLY index remained independently associated with 1-year mortality, along with EuroSCORE II and more than mild mitral regurgitation. Conclusions: The CALLY index is a strong and independent predictor of 1-year mortality after TAVI and provides incremental prognostic value beyond conventional surgical risk scores. Given its simplicity and reliance on routinely available laboratory parameters, the CALLY index may serve as a practical tool for long-term risk stratification in patients undergoing TAVI.

## 1. Introduction

Severe symptomatic aortic stenosis (AS) predominantly affects older adults and remains associated with relevant 1-year mortality even in the contemporary transcatheter era. Over the last decade, transcatheter aortic valve implantation (TAVI) has rapidly expanded from inoperable and high-surgical-risk patients to intermediate- and low-risk cohorts, supported by randomized trials and guideline recommendations [1,2,3]. In low-risk populations, randomized data show excellent short-term safety and sustained mid-term performance, while real-world studies still report appreciable 1-year all-cause mortality in the range of ~10–15%, underscoring residual risk beyond the valve intervention itself [4,5,6,7]. This gap motivates the refinement of prognostic assessment using parsimonious, biology-informed markers.

Traditional surgical risk scores, such as the Society of Thoracic Surgeons Predicted Risk of Mortality (STS score) and EuroSCORE II, were primarily developed to estimate perioperative risk, with a specific focus on in-hospital or 30-day mortality in patients undergoing cardiac surgery. Therefore, although they remain widely used in structural heart disease assessment, these scores may not adequately capture the long-term (e.g., 1-year) prognosis of contemporary TAVI candidates, whose outcomes are influenced not only by procedural risk but also by chronic inflammation, nutritional status, immune competence, frailty, and multimorbidity [3]. Recognizing these limitations, recent TAVI studies have increasingly highlighted the prognostic importance of pre-procedural biomarkers—particularly albumin, CRP, lymphocyte count, and renal function—as robust predictors of one-year mortality and adverse clinical events [4,6,7,8,9,10]. Beyond single markers, composite immunonutritional indices have gained traction in structural and coronary heart disease. In TAVI, the Naples Prognostic Score (NPS)—which integrates albumin, total cholesterol, neutrophil-to-lymphocyte ratio, and lymphocyte-to-monocyte ratio—has been reported as an independent predictor of 1-year mortality [10,11], supporting the concept that composite biology captures residual risk not addressed by valve replacement alone.

The C-reactive protein–albumin–lymphocyte (CALLY) index is a simple immunonutritional metric calculated as (albumin [g/L] × lymphocyte count [10^9^/L])/(CRP [mg/L] × 10), jointly reflecting systemic inflammation (CRP), nutritional status (albumin), and immune competence (lymphocytes). Prior cardiovascular studies—including acute coronary syndromes (ACS) and heart failure with preserved ejection fraction (HFpEF)—have linked lower CALLY values with higher mortality and adverse events, suggesting incremental prognostic information over isolated biomarkers [12,13,14,15,16].

However, to our knowledge, the prognostic value of the CALLY index has not yet been investigated in patients undergoing TAVI. Given the interplay between inflammation, malnutrition, and immune dysfunction in this frail population, the CALLY index may serve as a novel and simple biomarker to predict one-year mortality after TAVI. Therefore, this study aimed to assess the association between the baseline CALLY index and one-year all-cause mortality in patients undergoing TAVI and to compare its predictive performance with other established inflammatory and nutritional indices.

## 2. Materials and Methods

### 2.1. Study Design and Population

**Figure 1:**
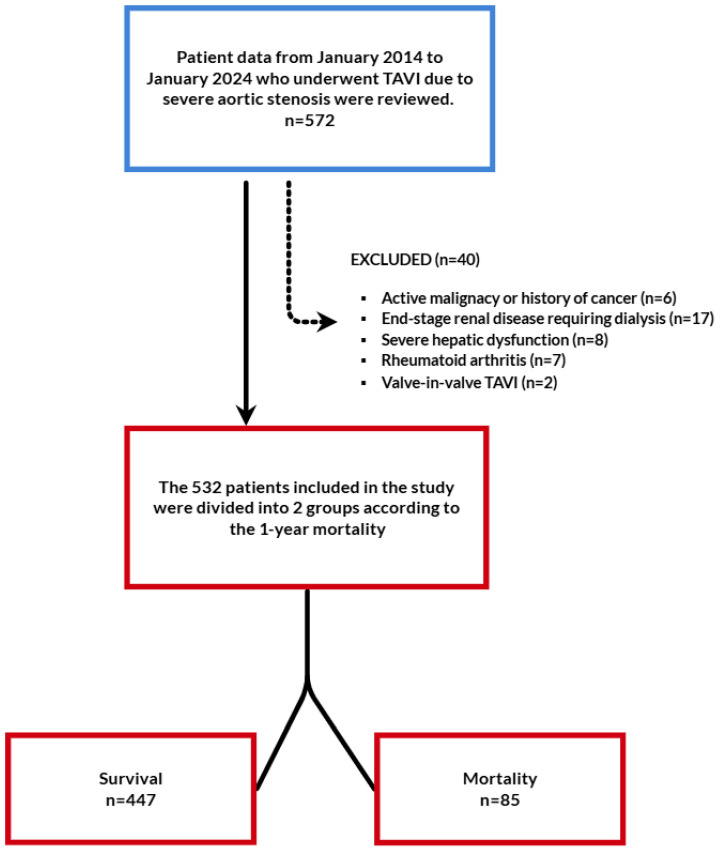
Flow chart of the study. TAVI: transcatheter aortic valve implantation.

This retrospective observational study was conducted in a tertiary-care cardiovascular center and included consecutive patients who underwent TAVI for severe symptomatic AS between January 2014 and January 2024. During the study period, a total of 572 patients underwent TAVI. After applying predefined exclusion criteria, 532 patients constituted the final study population. The patient selection process and exclusion criteria are summarized in Figure 1.

Patients were excluded if they had active infection or sepsis at the time of the index procedure, active malignancy, chronic kidney disease with an estimated glomerular filtration rate (eGFR) < 30 mL/min/1.73 m^2^, chronic liver disease classified as Child–Pugh class B or C, known autoimmune or systemic inflammatory diseases, valve-in-valve TAVI procedures, or severely incomplete medical records that precluded calculation of the CALLY index or assessment of outcomes.

Demographic characteristics, cardiovascular risk factors, comorbidities, laboratory parameters, echocardiographic findings, and procedural details were obtained from the institutional electronic medical records. The study was conducted in accordance with the principles of the Declaration of Helsinki and was approved by the Local Ethics Committee of Karamanoglu Mehmetbey University, Karaman, Turkey. Owing to the retrospective nature of the study, the requirement for written informed consent was waived.

### 2.2. TAVI Procedure

All patients were evaluated by a multidisciplinary Heart Team consisting of interventional cardiologists, cardiac surgeons, cardiac imaging specialists, and anesthesiologists. The decision to perform TAVI and the choice of valve type were made according to contemporary guideline recommendations and individual anatomical and clinical characteristics [17].

Preprocedural assessment included comprehensive transthoracic echocardiography to confirm the severity of aortic stenosis and evaluate left ventricular function, as well as multidetector computed tomography to assess aortic annular dimensions, vascular access routes, and calcification burden. Coronary angiography was performed according to institutional practice.

TAVI procedures were performed predominantly via transfemoral access under conscious sedation in accordance with institutional practice. Following vascular access, unfractionated heparin was administered to achieve an activated clotting time of >250 s. Balloon-expandable or self-expandable transcatheter heart valves were implanted under fluoroscopic guidance, based on anatomical characteristics and the Heart Team’s decision. Femoral access site hemostasis was achieved primarily using percutaneous vascular closure devices, while surgical cut-down was required in a small number of patients.

### 2.3. Laboratory Measurements and CALLY Index Calculation

Baseline blood samples were obtained within 24 h prior to the TAVI procedure as part of routine clinical evaluation. Complete blood count, serum biochemistry, and inflammatory markers were analyzed using standard automated laboratory methods.

For the purposes of this study, serum albumin concentration (g/L), C-reactive protein (CRP; mg/L), and absolute lymphocyte count (×10^9^/L) were recorded. The CALLY index was calculated using the following formula: CALLY index = (albumin (g/L) × lymphocyte count (10^9^/L))/(CRP (mg/L) × 10). Lower CALLY values reflect a higher burden of systemic inflammation, malnutrition, and immune dysregulation, whereas higher values indicate a more favorable immunonutritional status. Patients with active infection or sepsis were excluded from the study. In addition, the majority of patients underwent elective TAVI procedures, and prolonged preprocedural hospitalization or inter-hospital emergency transfer was uncommon in the study cohort, thereby limiting the potential impact of acute hospitalization-related inflammation on CRP levels.

### 2.4. Clinical Follow-Up and Outcomes

All patients were followed for a minimum of 12 months after the index TAVI procedure. Follow-up information was obtained through scheduled outpatient clinic visits, review of institutional electronic medical records, and telephone interviews when necessary. Mortality data were confirmed using hospital records and, when available, national death registries.

Clinical outcomes and procedural complications were defined and classified according to the Valve Academic Research Consortium-3 (VARC-3) criteria [18]. The primary outcome of the study was 1-year all-cause mortality following TAVI. Patients were categorized into survival and mortality groups based on their vital status at 1 year.

Secondary clinical outcomes included peri-procedural and in-hospital adverse events, such as vascular complications, conduction disturbances requiring permanent pacemaker implantation (PPMI), acute kidney injury (AKI), bleeding events, and procedural stroke. These events were systematically recorded during the index hospitalization and subsequent follow-up period in accordance with VARC-3 definitions.

### 2.5. Statistical Analysis

Statistical analyses were performed using IBM SPSS Statistics version 25.0 software (IBM Corporation, Armonk, NY, USA). The normality of continuous variables was assessed using skewness and kurtosis values, supported by visual inspection of histograms where appropriate. As the sample size exceeded 50 observations, distributions were considered approximately normal when skewness and kurtosis values were within ±2 [19]. Continuous variables with normal distribution were expressed as the mean ± standard deviation, whereas non-normally distributed variables were presented as the median with the interquartile range (25th–75th percentiles). Categorical variables were summarized as frequencies and percentages.

Comparisons between the survival and mortality groups were performed using the Student’s t test for normally distributed continuous variables and the Mann–Whitney U test for non-normally distributed variables. Categorical variables were compared using the Pearson χ^2^ test (Fisher’s exact test was applied when appropriate).

The discriminative ability of the CALLY index, EuroSCORE II, and STS score for predicting 1-year mortality was evaluated using receiver operating characteristic (ROC) curve analysis, and the area under the curve (AUC) was calculated. Optimal cut-off values were determined using Youden’s index. Based on the identified cut-off value, the CALLY index was also analyzed as a categorical variable.

To identify variables independently associated with 1-year mortality, univariable and multivariable regression analyses were performed. Variables with a *p* value < 0.10 in univariable analysis were entered into multivariable models. To avoid multicollinearity, individual components of the CALLY index (C-reactive protein, serum albumin, and lymphocyte count) were not included in multivariable analyses.

Two multivariable models were constructed: Model 1, in which the CALLY index was included as a continuous variable, and Model 2, in which the CALLY index was included as a categorical variable based on the ROC-derived cut-off value. Model calibration was assessed using the Hosmer–Lemeshow goodness-of-fit test, and model performance was evaluated using the Nagelkerke R^2^ statistic (Model 1: Hosmer–Lemeshow *p* = 0.573, Nagelkerke R^2^ = 0.226; Model 2: Hosmer–Lemeshow *p* = 0.434, Nagelkerke R^2^ = 0.309). The models demonstrated acceptable goodness of fit. All statistical tests were two-sided, and a *p* value <0.05 was considered statistically significant.

## 3. Results

A total of 532 consecutive patients with severe AS who underwent TAVI between January 2015 and January 2024 were included in this retrospective analysis. The overall one-year mortality rate was 15.9% (n = 85). Baseline demographic characteristics, comorbidities, and laboratory parameters of the study population are presented in Table 1. There were no significant differences between the survival and mortality groups with respect to age, sex, body mass index, or NYHA functional class. However, patients in the mortality group had significantly higher STS scores (7.07 ± 3.15 vs. 5.86 ± 3.11, *p* = 0.001) and EuroSCORE II values (median 11.7 vs. 7.8, *p* < 0.001).

Regarding comorbidities, the prevalence of atrial fibrillation (AF) (35.2% vs. 24.3%, *p* = 0.036) and previous myocardial infarction (MI) (18.8% vs. 10.5%, *p* = 0.030) was significantly higher in the mortality group. Other comorbid conditions were comparable between groups.

Significant differences were observed in several laboratory parameters. Patients who died within 1 year had higher neutrophil counts and C-reactive protein levels, and lower lymphocyte counts and serum albumin levels (all *p* < 0.05). Importantly, the CALLY index was markedly lower in the mortality group compared with survivors (median 0.36 vs. 0.82, *p* < 0.001). When categorized according to the ROC-derived cut-off value, CALLY index < 0.45 was significantly more frequent among patients who died during follow-up (71.7% vs. 25.0%, *p* < 0.001).

Echocardiographic and procedural characteristics are summarized in Table 2. Patients in the mortality group exhibited significantly lower left ventricular ejection fraction (LVEF) (46.5 ± 13.6% vs. 51.4 ± 13.5%, *p* = 0.002), higher systolic pulmonary artery pressure (sPAP) (48.2 ± 16.5 mmHg vs. 43.1 ± 17.1 mmHg, *p* = 0.010), and a higher prevalence of more than mild mitral regurgitation (MR) (50.5% vs. 31.3%, *p* = 0.001).

Procedural characteristics, including valve size, valve type, access route, and closure method, were similar between groups. The incidence of AKI was significantly higher in the mortality group (7.0% vs. 2.4%, *p* = 0.039), whereas rates of PPMI, major vascular complications, bleeding events, and procedural stroke did not differ significantly.

**Figure 2:**
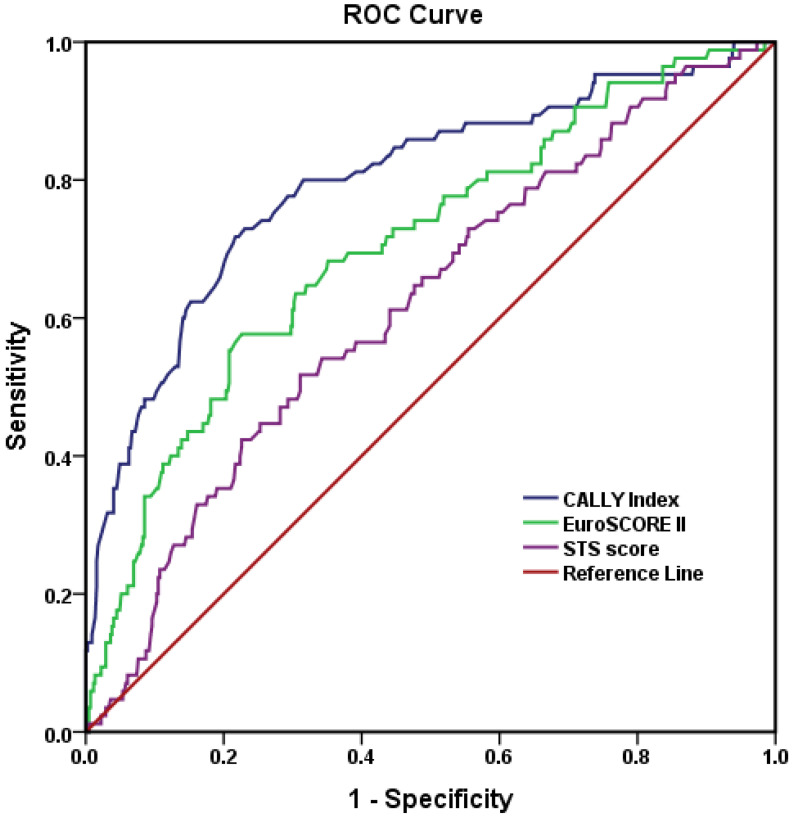
ROC curves of CALLY index, EuroSCORE II, and the Society of Thoracic Surgery risk score (STS score).

The ROC curve analysis demonstrated that the CALLY index had the highest discriminatory power for predicting one-year mortality after TAVI, with an AUC of 0.797 (95% CI: 0.740–0.854), which was higher than EuroSCORE II (AUC: 0.705; 95% CI: 0.643–0.767) and the STS score (AUC: 0.619; 95% CI: 0.555–0.683) (Table 3, Figure 2). Using the Youden index, the optimal cut-off value for the CALLY index was determined as 0.45, yielding a sensitivity of 71.8%, specificity of 78.3%, positive predictive value (PPV) of 38.4%, and negative predictive value (NPV) of 93.6%.

**Figure 3:**
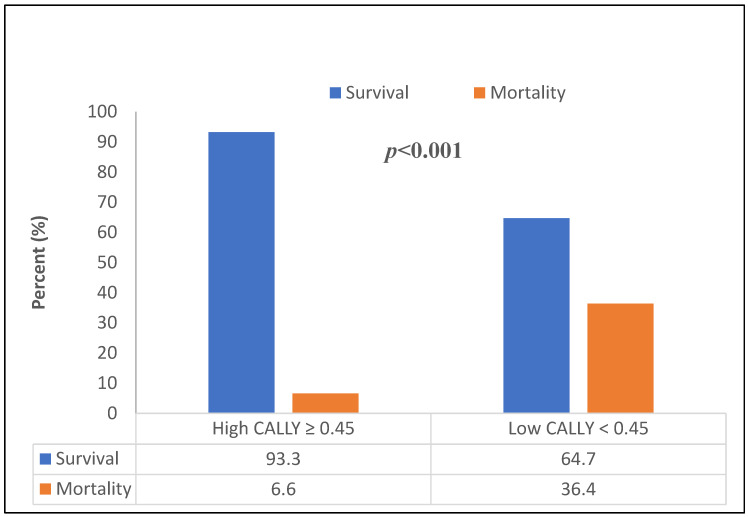
Survival and mortality distribution according to the CALLY index cut-off value (0.45) determined by the ROC curve with the Youden index. (Pearson’s χ^2^ test).

**Figure 4:**
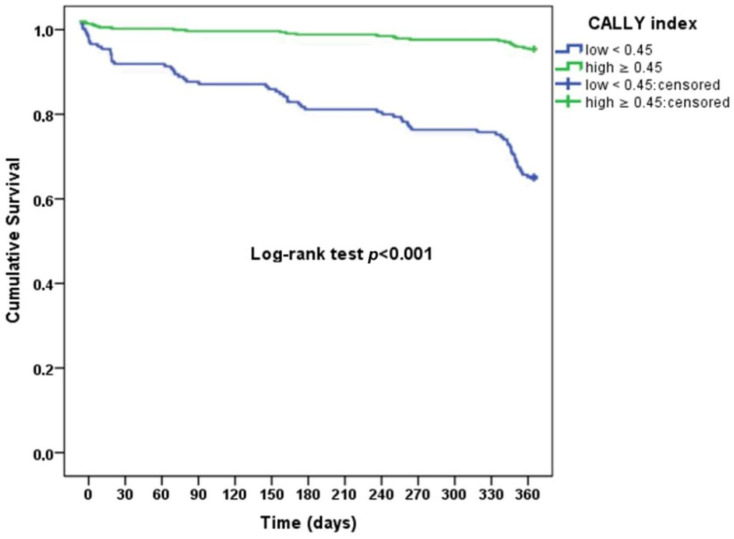
Kaplan–Meier survival plot showing marked divergence in survival curves among patients stratified by the CALLY index.

The distribution of survival and mortality according to the CALLY index cut-off value is illustrated in Figure 3. A significantly higher proportion of deaths was observed among patients with CALLY index < 0.45 (*p* < 0.001). Kaplan–Meier survival analysis further demonstrated a marked separation of survival curves between patients with low and high CALLY index values, with significantly reduced 1-year survival in the low CALLY group (log-rank *p* < 0.001; Figure 4).

In univariate logistic regression analysis, several variables including AF, neutrophil count, lymphocyte count, albumin, CRP, EuroSCORE II, STS score, LVEF, sPAP, more than mild MR, AKI, and both continuous and categorical CALLY indexes were associated with one-year mortality (Table 4).

Multivariate logistic regression analysis, adjusted for significant univariate predictors, identified the CALLY index as an independent predictor of mortality. When analyzed as a continuous variable (Model 1), each unit increase in the CALLY index was associated with a 26.5% reduction in mortality risk (OR: 0.735, 95% CI: 0.582–0.892, *p* < 0.001). Additionally, EuroSCORE II (OR: 1.088 per 1% increase, 95% CI: 1.042–1.127, *p* = 0.001) and more than mild MR (OR: 1.788, 95% CI: 1.031–3.105, *p* = 0.039) remained independent predictors in Model 1. When analyzed as a categorical variable (Model 2, CALLY < 0.45), a low CALLY index was associated with a 3.56-fold increased risk of one-year mortality (OR: 3.556, 95% CI: 2.123–5.636, *p* < 0.001). EuroSCORE II (OR: 1.065, 95% CI: 1.039–1.102, *p* = 0.001) and more than mild MR (OR: 1.875, 95% CI: 1.053–3.337, *p* = 0.033) also remained independent predictors in the categorical model.

## 4. Discussion

To the best of our knowledge, the present study is the first to evaluate the prognostic value of the CALLY index for 1-year mortality in patients undergoing TAVI. In a real-world cohort treated at a tertiary-care center, we demonstrated that a lower baseline CALLY index was strongly associated with increased 1-year mortality. Importantly, this association remained significant after adjustment for established clinical risk factors, echocardiographic parameters, and conventional surgical risk scores, underscoring the incremental prognostic value of this novel immunonutritional biomarker in the contemporary TAVI population.

The main findings of the present study can be summarized as follows: (i) patients who died within 1 year after TAVI had significantly lower baseline CALLY index values compared to survivors; (ii) the CALLY index demonstrated good discriminative ability for 1-year mortality and outperformed EuroSCORE II and the STS score in ROC analysis; and (iii) both continuous and categorical representations of the CALLY index were independently associated with mortality in multivariable models. Notably, patients with a CALLY index below the ROC-derived cut-off value experienced a more than threefold increased risk of death at 1 year, highlighting the clinical relevance of this marker for long-term risk stratification.

The prognostic significance of the CALLY index observed in our study is biologically plausible and aligns with accumulating evidence from other cardiovascular populations. The index integrates three key domains—systemic inflammation (CRP), nutritional status (albumin), and immune competence (lymphocyte count)—all of which have been individually linked to adverse outcomes in elderly and frail patients. Previous studies have demonstrated that a low CALLY index is associated with poor prognosis in ACS, HFpEF, and the no-reflow phenomenon in acute MI and has also been linked to an increased risk of adverse procedural outcomes, such as pocket hematoma formation after cardiac implantable electronic device implantation, suggesting that this composite biomarker reflects a global vulnerability phenotype rather than disease-specific risk alone [13,14,16,20]. Our findings extend this concept to the TAVI population, in whom chronic inflammation, malnutrition, and immune dysregulation are highly prevalent and may persist despite successful valve replacement. Indeed, systemic inflammation and hypoalbuminemia have repeatedly been implicated in adverse outcomes after TAVI, reflecting a complex interplay between inflammatory burden, impaired nutritional reserve, and immune dysfunction [21,22,23,24,25]. By integrating these interrelated pathophysiological domains, the CALLY index provides a more comprehensive reflection of patient vulnerability than isolated inflammatory or nutritional markers.

In the context of TAVI, conventional surgical risk scores such as the STS score and EuroSCORE II were primarily developed to estimate perioperative or short-term mortality and may not fully capture longer-term outcomes driven by patient-related systemic factors. Nevertheless, prior studies have demonstrated that these scores may retain prognostic value beyond the early period. In the OCEAN-TAVI registry, Ishizu et al. reported that the STS score was independently associated with long-term mortality after TAVI, supporting its potential role in extended risk stratification [26]. However, the discriminative performance of STS in that cohort remained modest. Similarly, Stähli et al. demonstrated that EuroSCORE II outperformed earlier risk models in predicting both early and late mortality after TAVI, whereas the STS score showed limited discriminatory capacity, with an AUC of approximately 0.55 [27]. These findings are consistent with the modest discriminative performance observed for STS in our cohort. In our study, EuroSCORE II remained an independent predictor of 1-year mortality in multivariable analysis, while the STS score did not. Nevertheless, although EuroSCORE II provided meaningful prognostic information, its discriminative performance for 1-year mortality was inferior to that of the CALLY index, highlighting the complementary value of immunonutritional assessment in long-term risk stratification after TAVI. Taken together, these findings suggest that while conventional surgical risk scores provide valuable baseline prognostic information, non-valvular and systemic factors, particularly those related to inflammation and nutritional status, play a pivotal role in determining longer-term outcomes after TAVI. This concept is increasingly supported by contemporary evidence demonstrating that composite inflammatory and immunonutritional biomarkers offer incremental prognostic value beyond traditional risk models. Indeed, similar observations have been reported with other composite indices, such as the NPS and systemic immune-inflammation-based indices, which have shown independent associations with 1-year mortality in TAVI cohorts [9,10,11,28]. In this context, the CALLY index appears to capture dimensions of patient vulnerability that are not fully reflected in surgical risk scores alone, thereby complementing established models for long-term risk stratification after TAVI.

An additional noteworthy finding of our study is that pre-existing AF was not independently associated with 1-year mortality after TAVI. This result is consistent with prior reports indicating that AF, although highly prevalent in the TAVI population, does not necessarily translate into increased early or mid-term mortality. In a contemporary cohort, Nso et al. demonstrated that both pre-existing and newly onset AF were not associated with increased 30-day mortality following TAVI, despite being linked to other adverse outcomes such as bleeding and rehospitalization [29]. Our findings extend these observations to a longer follow-up period, suggesting that AF per se may not be a dominant determinant of 1-year mortality when broader systemic risk factors are taken into account.

In a recent study by Güney et al., improvement in MR severity after TAVI was observed in a substantial proportion of patients; however, those with persistent moderate-to-severe MR experienced significantly higher mortality during follow-up [30]. Similarly, other observational studies have demonstrated that moderate-to-severe MR before or after TAVI is associated with increased mid- and long-term mortality, independent of procedural success [31,32]. In our cohort, the independent association between more than mild MR and 1-year mortality underscores the importance of comprehensive echocardiographic assessment beyond the aortic valve itself. Moreover, the coexistence of significant MR and a low CALLY index may reflect a particularly vulnerable phenotype characterized by advanced myocardial remodeling, chronic inflammation, and impaired nutritional reserve, which together contribute to adverse long-term outcomes after TAVI.

From a clinical perspective, the CALLY index provides a simple, inexpensive, and readily available tool for preprocedural risk stratification in patients undergoing TAVI. Beyond its association with increased mortality at low values, the relatively high negative predictive value of the CALLY index suggests that patients with preserved CALLY values may represent a subgroup with a favorable short- to mid-term prognosis. This feature is particularly relevant in daily practice, as it may help clinicians identify patients at lower risk for 1-year mortality, in whom standard follow-up strategies may be sufficient. Conversely, patients with a low CALLY index may benefit from closer clinical surveillance, optimization of nutritional and inflammatory status, and more individualized post-procedural management. Importantly, the CALLY index does not aim to replace established clinical or procedural risk scores but rather to complement existing models by capturing systemic vulnerability related to inflammation, nutrition, and immune dysregulation, dimensions that are not fully reflected in traditional risk stratification tools.

Several limitations of the present study should be acknowledged. First, this was a retrospective, single-center analysis, which may limit the generalizability of the findings. Second, although multiple clinically relevant confounders were adjusted for, residual confounding cannot be excluded. Third, the CALLY index was assessed only at baseline, and dynamic changes in inflammatory or nutritional status over time were not evaluated. Fourth, the low prevalence of malignancy in our cohort likely reflects patient selection, as individuals with active cancer were excluded, and advanced malignancy may influence referral patterns for TAVI in elderly patients. Finally, cause-specific mortality data were not consistently available; therefore, only all-cause mortality was analyzed. Prospective multicenter studies are warranted to validate these findings and to determine whether interventions targeting immunonutritional status can improve outcomes after TAVI.

## 5. Conclusions

In this real-world cohort of patients undergoing TAVI, the CALLY index was independently associated with 1-year mortality. In addition to the CALLY index, more than mild MR and EuroSCORE II were also independently associated with increased mortality, underscoring the combined impact of systemic patient vulnerability and underlying structural heart disease on long-term outcomes after TAVI. The CALLY index demonstrated superior discriminative ability for 1-year mortality compared with conventional surgical risk scores and remained robust after adjustment for established clinical, echocardiographic, and procedural risk factors.

Given its simplicity, low cost, and reliance on routinely available laboratory parameters, the CALLY index represents a practical immunonutritional biomarker for long-term risk stratification after TAVI. Incorporation of the CALLY index into clinical assessment may help identify high-risk patients who warrant closer follow-up as well as lower-risk individuals with a more favorable prognosis. Prospective multicenter studies are warranted to validate these findings and to determine whether interventions targeting immunonutritional status can improve outcomes in the expanding TAVI population.

## Figures and Tables

**Table 1:** Baseline demographic, comorbidity, and laboratory characteristics of the study population.

Variables	Overall (n = 532)	Survival (n = 447)	Mortality (n = 85)	*p*
Demographics				
Age (years)	77.6 ± 9.97	77.41 ± 8.05	78.57 ± 7.47	0.219 ^A^
Sex (female), n (%)	293 (55%)	241 (53.9%)	52 (61.1%)	0.236 ^B^
BMI (kg/m^2^)	26.4 (23.4–29)	26.4 (26.4–30)	26 (23–28.6)	0.422 ^C^
STS score (%)	6.0 ± 3.14	5.86 ± 3.11	7.07 ± 3.15	0.001 ^A^
EuroSCORE II (%)	8.4 (5.2–11.2)	7.8 (4.8–11.2)	11.7 (7.5–15.3)	<0.001 ^C^
NYHA, n (%)				
2	143 (26.8%)	122 (27.2%)	21 (24.7%)	0.649 ^B^
3	301 (56.5%)	254 (56.8%)	47 (55.2%)	
4	88 (16.5%)	71 (15.8%)	17 (20%)	
Comorbidities				
Diabetes mellitus, n (%)	156 (29.3%)	125 (27.9%)	31 (36.4%)	0.114 ^B^
Hypertension, n (%)	447 (84%)	373 (83.4%)	74 (87%)	0.405 ^B^
Hyperlipidemia, n (%)	270 (50.7%)	233 (52.1%)	37 (43.5%)	0.146 ^B^
Atrial fibrillation, n (%)	139 (26.1%)	109 (24.3%)	30 (35.2%)	0.036 ^B^
CAD, n (%)	386 (72.5%)	329 (73.6%)	57 (67%)	0.215 ^B^
Previous CABG, n (%)	128 (24%)	106 (23.7%)	22 (25.8%)	0.668 ^B^
Previous PCI, n (%)	187 (35.1%)	161 (36%)	26 (30.5)	0.337 ^B^
Previous MI, n (%)	63 (11.8%)	47 (10.5%)	16 (18.8%)	0.030 ^B^
COPD, n (%)	201 (37.7%)	166 (37.1%)	35 (41.1%)	0.481 ^B^
Bicuspid, n (%)	73 (13.7%)	66 (14.7%)	7 (8.2%)	0.162 ^B^
Stroke, n (%)	32 (6%)	28 (6.2%)	4 (4.7%)	0.760 ^B^
PAD, n (%)	42 (7.8%)	34 (7.6%)	8 (9.4%)	0.517 ^B^
Laboratory parameters				
Neutrophil (×10^3^/µL)	4.76 (3.6–6.1)	4.69 (3.6–6)	5.13 (4–6.4)	<0.001 ^C^
Lymphocyte (×10^3^/µL)	1.57 (1.13–2)	1.61 (1.16–2)	1.47 (1–1.93)	0.024 ^C^
Platelet (×10^3^/µL)	241.4 ± 82.6	238.1 ± 79.7	257.5 ± 95.7	0.056 ^A^
Hemoglobin (g/dL)	11.73 ± 1.9	11.77 ± 1.92	11.51 ± 1.79	0.267 ^A^
Albumin (g/L)	3.81 ± 0.35	3.83 ± 0.34	3.73 ± 0.40	0.021 ^A^
Creatinine (mg/dL)	1.12 ± 0.58	1.10 ± 0.59	1.18 ± 0.51	0.115 ^A^
GFR (ml/min/1.73 m^2^)	63.8 ± 20.32	64.38 ± 20	61.92 ± 21.31	0.286 ^A^
CRP (mg/L)	4.3 (2.39–7.88)	4.12 (2.29–7.6)	6.2 (3.5–9.74)	0.001 ^C^
TSH (μIU/mL)	1.30 (0.74–2.29)	1.33 (0.75–2.29)	1.29 (0.67–2.28)	0.583 ^C^
HbA1c (%)	6.51 ± 1.39	6.46 ± 1.37	6.75 ± 1.46	0.154 ^A^
Total cholesterol (mg/dL)	169.4 ± 44.1	170.6 ± 44.4	164 ± 42	0.153 ^A^
LDL (mg/dL)	100.5 ± 36.2	101.2 ± 36.1	97.4 ± 36.2	0.303 ^A^
CALLY index	0.73 (0.42–1.38)	0.82 (0.48–1.57)	0.36 (0.21–0.68)	<0.001 ^C^
CALLY index < 0.45, n(%)	173 (32.5%)	112 (25%)	61 (71.7%)	<0.001 ^B^

BMI: body mass index, CAD: coronary artery disease, COPD: chronic obstructive pulmonary disease, CRP: C-reactive protein, EuroSCORE II: the European System for Cardiac Operative Risk Evaluation, GFR: glomerular filtration rate, LDL: low-density lipoprotein, MI: myocardial infarction, NYHA: New York Heart Association, PAD: peripheral artery disease, STS: the Society of Thoracic Surgery risk score, and TSH: thyroid-stimulating hormone. Continuous variables are shown as the mean ± SD or median (25th–75th) percentiles where appropriate. ^A^ Student’s *t* test, ^B^ Pearson’s χ^2^ test, and ^C^ Mann–Whitney U test. Note: The CALLY index cut-off value (0.45) was determined by receiver operating characteristic (ROC) curve analysis using Youden’s index for the prediction of 1-year mortality.

**Table 2:** Echocardiography and procedural characteristics of the study population.

Variables	Overall (n = 532)	Survival (n = 447)	Mortality (n = 85)	*p*
Echocardiography				
LVEF (%)	50.58 ± 13.63	51.36 ± 13.51	46.47 ± 13.55	0.002 ^A^
LA diameter (cm)	4.67 ± 0.65	4.65 ± 0.65	4.76 ± 0.61	0.192 ^A^
Aortic max gradient (mmHg)	79 (67–94)	78.5 (67–94)	79 (67–93)	0.940 ^C^
Aortic mean gradient (mmHg)	47 (41–58)	47 (41–59)	47 (41–55)	0.772 ^C^
AVA (cm^2^)	0.67 ± 0.16	0.67 ± 0.16	0.64 ± 0.16	0.178 ^A^
Aortic annulus (cm)	2.15 ± 0.20	2.15 ± 0.20	2.13 ± 0.21	0.303 ^A^
sPAP (mmHg)	43.89 ± 17.05	43.06 ± 17.05	48.24 ± 16.49	0.01 ^A^
More than mild MR, n (%)	183 (34.3%)	140 (31.3%)	43 (50.5%)	0.001 ^B^
AS group, n (%)				
High gradient	350 (65.7%)	298 (66.6%)	52 (61.1%)	0.287 ^B^
LF–LG	39 (7.3%)	30 (6.7%)	9 (10.5%)	
Paradoxal LF–LG	10 (1.8%)	7 (1.5%)	3 (3.5%)	
Very severe AS	133 (25%)	112 (25%)	21 (24.7%)	
Procedural characteristics				
Valve size (mm), n (%)				
20	2 (0.3%)	2 (0.4%)	–	0.780 ^B^
23	224 (42.1%)	183 (40.9%)	41 (48.2%)	
25	14 (2.6%)	12 (2.6%)	2 (2.3%)	
26	215 (40.4%)	186 (41.6%)	29 (34.1%)	
27	6 (1.1%)	5 (1.1%)	1 (1.1%)	
29	71 (13.3%)	59 (13.1%)	12 (14.1%)	
Valve in valve	7 (1.3%)	5 (1.1%)	2 (2.3%)	0.311 ^B^
Closure method, n (%)				
ProGlide	325 (61%)	272 (60.8%)	53 (62.3%)	0.961 ^B^
Prostar	176 (33%)	149 (33.3%)	27 (31.7%)	
Cut-down	31 (5.8%)	26 (5.8%)	5 (5.8%)	
Edwards SAPIEN XT, n (%)	457 (85.9%)	387 (86.5%)	70 (82.3%)	0.309 ^B^
Edwards SAPIEN 3, n (%)	43 (8%)	34 (7.6%)	9 (10.5%)	0.384 ^B^
LOTUS valve, n(%)	24 (4.5%)	19 (4.2%)	5 (5.8%)	0.566 ^B^
ACURATE neo, n (%)	8 (1.5%)	7 (1.5%)	1 (%1.1)	0.815 ^B^
Transaxillary access, n (%)	20 (3.7%)	17 (3.8%)	3 (3.5%)	0.953 ^B^
Post-TAVI pacemaker, n (%)	48 (9%)	39 (8.7%)	9 (10.5%)	0.540 ^B^
Procedural stroke, n (%)	4 (0.7%)	2 (0.4%)	2 (2.3%)	0.122 ^B^
Major vascular complication, n (%)	23 (4.3%)	19 (4.2%)	4 (4.7%)	0.896 ^B^
Life-threatening bleeding, n (%)	4 (0.7%)	3 (0.6%)	1 (1.1%)	0.685 ^B^
Acute kidney injury, n (%)	17 (3.1%)	11 (2.4%)	6 (7%)	0.039 ^B^

AVA: aortic valve area, AS: aortic stenosis, LA: left atrium, LF–LG: low flow–low gradient, LVEF: left ventricular ejection fraction, sPAP: systolic pulmonary artery pressure, and TAVI: transcatheter aortic valve implantation. Continuous variables are shown as the mean ± SD or median (25th–75th) percentiles where appropriate. ^A^ Student’s *t* test, ^B^ Pearson’s χ^2^ test (Fisher’s exact test where appropriate), and ^C^ Mann–Whitney U test.

**Table 3:** ROC curve analyses of the CALLY index, EuroSCORE II, and STS score for 1-year mortality after TAVI.

Variable	AUC (%95)	*p*	Cut-off	Sensitivity (%)	Specificity (%)	PPV (%)	NPV (%)
CALLY	0.797 (0.740–0.854)	<0.001	0.45	0.718	0.783	38.4	93.6
EuroSCORE II	0.705 (0.643–0.767)	<0.001	11.27	0.637	0.765	32.7	90.6
STS score	0.619 (0.555–0.683)	0.001	6.82	0.518	0.689	24	88.3

AUC: area under the curve, EuroSCORE II: the European System for Cardiac Operative Risk Evaluation, NPV: negative predictive value, PPV: positive predictive value, ROC: receiver operating characteristic, STS: the Society of Thoracic Surgery risk score, and TAVI: transcatheter aortic valve implantation.

**Table 4:** Independent predictors of 1-year mortality after TAVI by logistic regression analysis.

Variables	Univariate Analysis		Multivariate Analysis—Model 1 *		Multivariate Analysis—Model 2 **	
	OR (95%CI)	*p*-Value	OR (95%CI)	*p*-Value	OR (95%CI)	*p*-Value
Age	1.019 (0.989–1.050)	0.219	-	-	-	-
DM	1.479 (0.908–2.408)	0.116	-	-	-	-
AF	1.691 (1.032–2.773)	0.037	1.176 (0.675–2.048)	0.567	1.221 (0.687–2.169)	0.497
Albumin	0.463 (0.240–0.894)	0.022	-	-	-	-
CRP	1.022 (0.999–1.045)	0.057	-	-	-	-
Neutrophil	1.158 (1.046–1.282)	0.005	1.059 (0.936–1.198)	0.365	1.068 (0.936–1.218)	0.330
Lymphocyte	0.634 (0.433–0.928)	0.019	-	-	-	-
Platelet	1.003 (1.000–1.005)	0.058	1.003 (0.999–1.006)	0.101	1.002 (0.999–1.005)	0.236
Previous MI	1.973 (1.059–3.677)	0.032	1.282 (0.610–2.695)	0.513	1.436 (0.664–3.108)	0.358
STS score	1.12 (1.044–1.22)	0.001	0.992 (0.904–1.088)	0.786	0.993 (0.899–1.095)	0.885
EuroSCORE II	1.103 (1.065–1.162)	<0.001	1.088 (1.042–1.127)	0.001	1.065 (1.039–1.102)	0.001
* CALLY index	0.454 (0.256–0.715)	<0.001	0.735 (0.582–0.892)	<0.001	-	-
** CALLY index < 0.45	5.579 (3.146–9.636)	<0.001	-	-	3.556 (2.123–5.636)	<0.001
LVEF (%)	0.976 (0.961–0.992)	0.003	0.991 (0.972–1.010)	0.353	0.993 (0.973–1.013)	0.498
sPAP	1.017 (1.004–1.031)	0.011	1.004 (0.989–1.020)	0.582	1.004 (0.988–1.021)	0.606
More than mild MR	2.245 (1.403–3.592)	0.001	1.788 (1.031–3.105)	0.039	1.875 (1.053–3.337)	0.033
AKI	3.010 (1.082–6.865)	0.035	2.970 (0.981–5.994)	0.054	1.514 (0.492–2.963)	0.470

AF: atrial fibrillation, AKI: acute kidney injury, CI: confidence interval, CRP: C-reactive protein, DM: diabetes mellitus, LVEF: left ventricle ejection fraction, MI: myocardial infarction, MR: mitral regurgitation, OR: odds ratio, sPAP: systolic pulmonary artery pressure, STS: the Society of Thoracic Surgery risk score, and TAVI: transcatheter aortic valve implantation. * CALLY index presented as a continuous variable; ** CALLY index < 0.45 presented as a categorical variable. Note: Albumin, lymphocyte, and CRP were not included in multivariate models to avoid multicollinearity with the CALLY index.

## Data Availability

No material from other sources was reproduced in this manuscript. All content is original. The data presented in this study are available on request from the corresponding author. The data are not publicly available due to privacy and ethical restrictions.
